# Evaluating Coaching Intervention for Financial Risk Perception and Credit Risk Management in a Nigerian Sample

**DOI:** 10.3389/fpsyg.2022.962855

**Published:** 2022-07-05

**Authors:** Robinson Onuora Ugwoke, Edith Ogomegbunam Onyeanu, Obioma Vivian Ugwoke, Tijani Ahmed Ajayi

**Affiliations:** Department of Accountancy, Faculty of Business Administration, University of Nigeria Enugu Campus, Nsukka, Nigeria

**Keywords:** business coaching, coaching, credit risk management, financial risk perception, REBT

## Abstract

There is no doubt that a negative perception of financial risk and a lack of credit risk management adversely impact business growth and business owners’ wellbeing. Past studies suggest that most Nigerian traders have poor risk perceptions and manage risk poorly. A business coaching program within rational-emotive behavior therapy framework (REBT-based business coaching) was evaluated in order to determine its effects on financial risk perception and credit risk management among Nigerian traders. This study used an open-label parallel randomized control design. This 8-weeks coaching program had 60 traders in the REBT-based business coaching group and 59 traders in the control group. The mixed-model repeated measures ANOVA was utilized for analysis of the study data. Results show that relative to a control group, traders’ financial risk perception [*F*_(1.09,127.15)_ = 637.29, *p* < 0.001, ω^2^ = 0.69] and credit risk management [*F*_(1.55,181.22)_ = 795.51, *p* < 0.001, ω^2^ = 0.80] significantly improved after participating in REBT-based business coaching program. This study shows that REBT-based business coaching program is integral to increasing financial risk perception and credit risk management among Nigerian traders. This study contributes to the advancement of business coaching program within the rational-emotive behavior therapy framework for market traders, and its application in similar situations. The study considered the benefits of business coaching program for market traders from a developing country, which is a rarely studied group. It is highly recommended that professionals study the relationship between REBT-based business coaching and economic decision-making within various organizational contexts.

## Introduction

In the business world, risk refers to the possibility of losing money invested in a business venture (Willet, 2002). The likelihood of incurring a loss on a business venture is referred to as financial risk, according to Hayes (2021). Financial risk perception refers to the way a person think, feel, and evaluates the risk factors of an ongoing or potential business venture (Nguyen et al., 2019). Often, financial risk perception studies employ psychometric scaling techniques in order to generate quantitative data about how people think about risk and benefit (e.g., Diacon and Ennew, 2001; Nguyen et al., 2019; Tavares et al., 2020). Credit risk is a term that describes the likelihood of loss due to the failure to comply with the terms of a financial contract (Brock, 2021; Corporate Finance Institute, 2022; SAS Institute, 2022). Default on a loan is a common example of credit risk (Brock, 2021; Corporate Finance Institute, 2022). On the other hand, credit risk management refers to the process of evaluating a business owner’s capital position and credit rating in order to assist them to mitigate losses and prevent liquidation of the business (Corporate Finance Institute, 2022).

There is no doubt that a negative perception of financial risk and a lack of credit risk management adversely impact business growth and business owners’ wellbeing (Odetokun et al., 2018; Aderonke et al., 2021). Nigerian market traders are not exempt from the financial losses experienced by many business owners and traders (Alonso, 2020). When it comes to doing business within Nigeria, traders are often faced with a range of problems and roadblocks that adversely affect their mental health and wellbeing (Gbandi and Amissah, 2014; Odetokun et al., 2018). A growing number of small- and medium-scale Nigerian traders have expressed concern about the lack of financing that causes them to fail to sustain their businesses after some time (Gbandi and Amissah, 2014). According to a previous report by Gbandi and Amissah (2014), financial concerns negatively impacted 39% of small-scale traders and 37% of medium-scale traders in Nigeria. Most Nigerian traders own and operate small and medium-sized businesses making it crucial to enhance their awareness of financial risks and credit risk management in order to avoid financial distress (Mbah et al., 2016).

Most Nigerian traders appear to have poor risk perception and manage risk poorly, according to studies by Odetokun et al. (2018) and Aderonke et al. (2021). In their study, Aderonke et al. (2021) identified 34.7 percent of the traders who have poor attitudes toward risk and 21 percent who have poor perception of risk. A report also claims that negative perceptions of risk and prejudice undermine the success of traders on the African continent, including Nigerian traders (Alonso, 2020). Small and medium-sized Nigerian traders may benefit from research on how to enhance their perception of financial risk and credit risk management. A business owner who understands economic and credit risks and handles them cautiously may be able to boost productivity and wellbeing (Lammers et al., 2010). In this research, we propose to study the effect of coaching on financial risk perception and credit risk management among Nigerian market traders. Whereas experts are diligently searching for evidence-based strategies to offer effective financial and debt counseling to their diverse clientele, it appears that coaching is underappreciated in different business contexts as a tool for improving credit risk perception and management. This study’s aim is to evaluate the effect of a business coaching program within rational-emotive behavior therapy framework (REBT-based coaching) on Nigerian market traders’ financial risk perception and credit risk management. Accordingly, we hypothesized that a REBT-based business coaching program would improve traders’ perception of financial risk and ability to manage credit risk.

Coaching within the business world requires a deep understanding of financial risk perception and management (Onyeanu et al., 2022). The coaching process involves providing support, advice, and tutelage to a person or group (Zarate, 2002). In coaching, individuals are guided toward achieving set objectives, and they are often evaluated as the goals are reached (Zuñiga-Collazos et al., 2020). Business coaching is a concept used to refer to the process that drives a business from a certain state to where the owner desires it to be by enabling the coach to assist the owner in clarifying the business vision and how it suits their individual goals (The Alternative Board, 2015). Seasoned entrepreneurs who teach other entrepreneurs how to build and grow their own businesses are most often known as business coaches (Brian, 2022). The initial step in the business coaching process is to recognize the direction in which a business owner wishes to take their enterprise (The Alternative Board, 2015; Brian, 2022). Business coaches then assist in planning and prioritizing the goals and strategies that will assist in moving the enterprise toward its ultimate goal (The Alternative Board, 2015; Brian, 2022). Coaches may meet with business owners on a weekly or monthly basis to ensure that they are adhering to the commitments made during their previous coaching sessions (The Alternative Board, 2015). Business coaching involves a high degree of accountability and it is the responsibility of coaches to keep track of goals by developing key performance indicators that make the process of achieving them as transparent as possible (The Alternative Board, 2015). Based on a recent survey by Wiginton and Cartwright (2020), respondents were very confident that business coaching contributes both to their own growth and to their business’ success as well.

As with most rational emotive behavioral therapy (REBT) methods (Ellis, 1994; Anderson, 2002), REBT-based business coaching may aid in improving financial risk perception and the management of risk in business situations. In REBT-based business coaching, REBT’s dimensions are adapted to business coaching contexts. REBT-based business coaching is a psychosocial risk management program designed to increase perceptions of financial risk and management in business situations. This program model is influenced by cognitive-behavioral coaching models (e.g., Kodish, 2002; Otu, 2020; Onyeanu et al., 2022), and seeks to modify the coachees’ dysfunctional beliefs, attitudes, thoughts, and behaviors concerning financial risk and management. A REBT-based coaching intervention can be provided face-to-face, in a group setting, through a smartphone application, or by e-mail (Ogbuanya et al., 2017; Ezenwaji et al., 2019). A REBT-based intervention is often predicated on the idea that how individuals recognize and consider events strongly affect how they feel and react to them (Ellis, 1956; Neenan and Dryden, 2002; Katsikis et al., 2016; David and Bernard, 2018; Ogba et al., 2020; Ene et al., 2021). Thus, in REBT-based business coaching, coaches can capitalize on a model of thoughts and behavior modification to direct traders to make rational efforts to improve their ability to enhance their financial risk perception and credit risk management and to achieve a greater understanding of monetary knowledge to be used within the present and future scenarios.

The REBT-based coaching model has demonstrated effectiveness in managing dysfunctional cognitions, feelings, and behaviors and may assist market traders in managing unhelpful credit risk and perceptions of risk. For instance, in the treatment of work stress and the decrease of irrational beliefs, REBT-based coaching intervention was significantly effective (Ogbuanya et al., 2017; Ogba et al., 2019; Ugwuanyi et al., 2021). Work-life quality is improved by REBT-based coaching (Agu et al., 2021). According to Otu and Omeje (2021), REBT-based coaching significantly improved illogical career beliefs. Nwokeoma et al. (2019) and Onyishi et al. (2021) found that coachees’ subjective well-being and feelings were positively affected by REBT-based coaching. REBT-based coaching research showed that participants can better manage depression after exposure to it (Eseadi et al., 2017a,b). Participants in Ezenwaji et al.’s (2019) study were able to cope with burnout issues through REBT-based coaching. To date, however, no REBT-based coaching program has been developed for improving financial risk perception and credit risk management among market traders. In order to fill this gap, this study was undertaken. In other words, the purpose of this research was to evaluate the effect of REBT-based coaching on financial risk perception and credit risk management among Nigerian market traders.

## Method

### Study Setting

Anambra State, located in southern Nigeria, was the setting for the study. Historically, the state has been a port for trade and commerce. As a result of the Onitsha market in Onitsha, Anambra State has the biggest commercial market in southeast Nigeria. The Onitsha market is a renowned market in Nigeria (Onyeakagbu, 2021). The market is rightly referred to as West Africa’s commercial powerhouse and is frequented by merchants from around the region (MyCostoma, 2021). In terms of services, culture, and product diversity, the Onitsha marketplaces are different from other markets around the world.

### Study Participants

Participants in the study were traders in Nigeria’s Onitsha main market. The traders were selected for the eligibility assessment by using simple random sampling methods. The final sample size was determined (*d* = 0.50, 80% power) through the G*power 3.1.1 sample size estimation software (Faul et al., 2007). The study was completed by 60 traders in the REBT-based business coaching group and 59 traders in the control group. The maximum number of small groups was six as each group had between nine and ten traders. We assigned 60 participants to the coaching group and 59 participants to the control group by random numbers generated using the Random Allocation Software (RAS) program (Saghaei, 2004). According to Beene (2020) and Sutton (2022), approximately 10–15 participants are adequate for a group coaching program.

### Data Collection

A variety of demographic information was collected in this study about traders, including their gender, marital status, age, level of education, and years as traders. The Traders’ Financial Risk Perception Questionnaire (TFRPQ) was adapted from previous literature and used as one of the primary instruments of data collection (Nguyen et al., 2019; Tavares et al., 2020). With a 5-point Likert scale [strongly agree (1) to strongly disagree (5)], the TFRPQ measures traders’ feelings about financial risks using five items. Example of item in the TFRPQ include: I live in fear of losing money whenever I invest. Cronbach’s alpha for the questionnaire was 0.74.

The Traders’ Credit Risk Management Questionnaire (TCRMQ) is another instrument used to collect data. It was developed based on previous literature (Lhabitant and Tinguely, 2001; Horcher, 2005; Belás et al., 2015). With a 5-point response scale [strongly agree (1) to strongly disagree (5)], the TCRMQ measures traders’ credit risk management using five items. An example of an item in the TCRMQ is: I feel awful a lot about my credit score, I cannot stand it. According to Cronbach’s alpha, the questionnaire had an internal consistency reliability of 0.72.

### Inclusion and Exclusion Criteria

Participants were asked to consent in writing to participate in the study as a first criterion for inclusion. The second criterion involved potential participants meeting the cut-off scores for poor financial risk perception and poor credit management. As a third criterion, potential participants had to have a history of ongoing loan obtained to support their businesses (the aim is to ensure that research participants have a grasp of how and why credit management is essential). Potential participants were excluded from the study if they did not meet these criteria.

### Treatment Procedure

As in the treatment group, the traders in the control group received treatment-as-usual for the same length of time. Traders who receive treatment-as-usual receive conventional business counseling that do not specifically address a certain issue for an extended period of time. Every meeting day is devoted to a different business topic which members of the group discuss and analyze over the course of 8 weeks.

REBT-based business coaching was given to the treatment group. REBT-based business coaching program meetings were held twice a week from 4 to 5 p.m. for 8 weeks (and there was a 2-week follow-up period after the program ended). Based on Otu’s (2020) and Onyeanu et al.’s (2022), REBT coaching manual, we developed the coaching intervention program. The traders participated in the program *via* telegram and email to improve their understanding of financial risk and credit risk management. In order to ensure that coaching participants met their goals for joining the program, cognitive, behavioral, emotive, and problem-solving tools were included in the program. Also included in REBT coaching techniques are alliance design, values identification, homework, the future self, goal setting, dating, and the big “A” agenda (Kodish, 2002). The treatment sessions are below.

The first and second weeks of the program included introductions, familiarizations, REBT-based business coaching expectations, briefings regarding financial risk perception and credit risk management, and explanations of the various aspects of the coaching program. The third and fourth weeks were devoted to applying the coaching techniques to improve financial risk perception and credit risk management. As part of these weeks, coaches explored participants’ eagerness to modify their perceptions of financial risk and to build credit risk management strategies, as well as their potential commitments to improving their perceptions of financial risk.

Through the 5th and 6th weeks, participants learned more about how to perceive financial risk and how to manage it effectively. Through several coaching tasks, participants were equipped emotionally, cognitively, and behaviorally to identify and apply rational strategies for maintaining a positive financial risk perception and managing credit risk effectively. Participants at these stages were prepared to become their own REBT-based business coaches and encouraged to use the skills they acquired in the coaching program in the future.

Seventh and eighth weeks were devoted to follow-up in order to assess the persistence of goals achieved during the coaching program, centered around positive financial risk perception and effective credit risk management.

Four business coaches (two males and two females) with at least 3 years postdoctoral training experience delivered the interventions to the participants. Due to their experience in applying REBT in the context of business coaching, these coaches were considered suitable for this study. Two independent business coaches supervised the coaches’ compliance with the treatment recommendations alongside the researchers.

### Treatment Fidelity, Adherence, and Monitoring

We monitored participants’ participation in the intervention sessions by recording their weekly attendance, asking them questions about each session and keeping track of how they completed them. By observing coaches’ adherence to coaching components, and tracking the time spent, six external raters used a treatment integrity rating scale to get a holistic view of the coaching implementation. Further, a *post hoc* treatment fidelity check was completed using the procedure of Borrelli et al. (2005). Coaches were required to assess participants’ performances on a weekly basis to also monitor how they implemented sessions.

### Controlling of Attrition

Make-up sessions and breakout sessions were offered on a weekly basis before the next meeting to participants who missed sessions. Participants were given this opportunity in order to prevent attrition. There was also little financial incentive ($10.00) to cover the costs of transportation and refreshments during treatment sessions.

### Study Design and Ethics

This study was conducted between May and October of 2021, and it used open-label, parallel randomized control trial design. This is a true experimental design which is used for group interventions (Lee et al., 2009). University of Nigeria’s Committee on Research Ethics approved the study. The study was conducted according to the research guidelines of the American Psychological Association.

### Data Analysis

In order to address the issue of introducing statistical dependencies into group-based therapy data, Baldwin et al. (2008) suggested the use of mixed-model repeated measures ANOVA for analyzing the research data. This repeated measures model considers the interaction between Time and Condition as a statistical test for intervention effects. This interaction is used in order to examine whether the intervention group has a greater level of variation in the dependent variable than the control group (Baldwin et al., 2008). They propose analyzing the data based on two models, in which the first model evaluates treatment effects without reference to a group (w/o group), whereas the second model evaluates treatment effects based on a group (w/group) while taking Time into account as a categorical variable. The authors suggest using omega squared (ω^2^) for both models and calculating variance inflation factor (VIF) for both models to evaluate inflation. In accordance with Baldwin et al. (2008), intraclass correlation coefficients (ICCs) were determined. In order to understand the ICC, Kim et al. (2006) recommended comparing the magnitude of the ICC with ω^2^ from the model including group. Sphericity correction was done based on Greenhouse-Geisser, because the Mauchly’s test indicates that the assumption was violated. The data were processed and analyzed using JASP 0.16 (University of Amsterdam) and Statistical Package for the Social Sciences, ver. 22.

## Results and Discussion

Data in Table 1 show the following demographic categories for participants in each group: education level, marital status, years of experience trading, and gender. The descriptive statistics are presented in Table 2 for the outcomes of each intervention. Furthermore, the results for first model (group excluded) indicate that the REBT-based business coaching program had a significant positive effect on traders’ financial risk perception, *F*_(1.01,119.54)_ = 237.60, *p* < 0.001, ω^2^ = 0.41. Additionally, in the second model (group included), the REBT-based business coaching program also produced significant positive effects on market traders’ financial risk perception, *F*_(1.09,127.15)_ = 637.29, *p* < 0.001, ω^2^ = 0.69. Also the results for the first model (group excluded) demonstrate that the REBT-based business coaching program resulted in significant positive impacts on the traders’ credit risk management, *F*_(1.06, 125.44)_ = 172.30, *p* < 0.001, ω^2^ = 0.34. The second model (group included) also confirmed that the REBT-based business coaching program had a significant positive impact on the traders’ credit risk management, *F*_(1.55,181.22)_ = 795.51, *p* < 0.001, ω^2^ = 0.80. It was found that the ω^2^ (0.69) was greater than the ICC (0.48) in terms of market traders’ perceptions of financial risk. In relation to credit risk management, market traders’ ω^2^ (0.80) was greater than their ICC (0.47). According to these findings, the REBT-based business coaching program contributed to the significant improvements observed in market traders’ perceptions of financial risk and credit risk management.

**TABLE 1:** Demographic profile of the participants by intervention conditions.

1	Demographics of participants	Coaching group, n (%)	Control group, n (%)
Education level	Primary Education	18 (30.0)	17 (28.8)
	Secondary Education	13 (21.7)	15 (25.4)
	Diploma	16 (26.7)	16 (27.1)
	Degree	13 (21.7)	11 (18.6)
Age	20–30 years	28 (46.7)	28 (47.4)
	31–40 years	23 (38.3)	21 (35.6)
	Above 40 years	9 (15.0)	11 (18.6)
	Single	17 (28.3)	14 (23.7)
Marital status	Married	20 (33.3)	19 (32.2)
	Divorced	10 (16.7)	11 (18.6)
	Separated	13 (21.7)	15 (25.4)
Years of experience as a trader	1–5 years	8 (13.3)	12 (20.3)
	6–15 years	17 (28.3)	19 (32.2)
	16–25 years	26 (43.3)	24 (40.7)
	Above 25 years	9 (15.0)	4 (6.8)
Gender	Male	34 (56.7)	32 (54.2)
	Female	26 (43.3)	27 (45.8)

**TABLE 2:** Descriptive statistics for the outcome measures by intervention conditions and time.

		TFRPQ		TCRMQ	
			
Time	Intervention conditions	Mean	SD	Mean	SD
Time 1	Control group	9.58	0.99	6.63	1.39
	Coaching group	9.53	1.02	6.58	1.32
Time 2	Control group	12.10	1.77	7.95	0.94
	Coaching group	21.40	1.64	17.80	1.01
Time 3	Control group	12.10	1.77	8.12	1.10
	Coaching group	21.58	1.64	17.88	1.08

*TFRPQ, Traders’ Financial Risk Perception Questionnaire; TCRMQ, Traders’ Credit Risk Management Questionnaire; SD, Standard Deviation.*

Based on our study, traders showed significant improvements in credit risk management after receiving REBT-based business coaching. This study confirms previous finding that REBT-based coaching can lead to significant improvements in participants’ risk management, as reported by Ogbuanya et al. (2017). This study also corroborates the finding that REBT-based coaching can result in significant improvements in how participants view and tolerate financial risk, as reported by Onyeanu et al. (2022). Our findings corroborate previous studies that have found that REBT-based interventions can assist participants in altering negative attitudes and managing perceived risks (Onyechi et al., 2016; Roberts, 2017; Ofoegbu et al., 2020). This study suggests that REBT-based business coaching can be an effective intervention in improving financial risk perception and credit risk management among Nigerian traders. REBT-based interventions have the ability to identify and correct participants’ inaccurate perceptions, according to Bernard (2016). Another study suggests the use of REBT-based coaching benefits clients (Nwokeoma et al., 2019). Considering that this is a preliminary investigation into Nigerian traders’ perceptions of financial risk and credit risk management using REBT-based coaching, more investigation is needed to better comprehend the impacts of financial risk perceptions and credit risk management on Nigerian traders.

### Study Limitations

The research was limited to the main market traders of Onitsha. This means that it cannot be generalized to all market traders in the country. The moderators of financial risk perception and credit risk management should be studied in a prospective study using key demographic variables such as culture, race, and religion. The REBT-based business coaching program was evaluated solely with quantitative data. There is a need for further studies using qualitative data paradigms to investigate the impact of REBT-based business coaching programs in other business groups such as online market traders and student-entrepreneurs. One of the limitations of our study was also the lack of a satisfaction measure for participants with REBT-based business coaching. It is suggested that future research in this area study the satisfaction of participants with REBT-based business coaching. As part of our re-examination of the effectiveness of the REBT-based business coaching, we also hope to examine this in any future research.

### Practical Implications and Suggestions

In the context of business coaching, market research and behavioral accounting research, this study has practical implications. This study may be useful for business coaches, behavioral accounting professionals and their teams to better understand how clients’ perceptions of financial risk and credit risk management impact their business growth as well as possible behavioral intervention strategies that can be tailored to assist them during service delivery. The insights from this research can also be used by policymakers to improve business conditions for small- and medium-sized traders, as well as to synergize existing programs aimed at this population. Researchers could also use the insights from this study to study the relationship between business coaching and economic decision-making within various organizational contexts. Business coaching based on REBT framework can greatly benefit accounting clients like traders, since cognitive processes and perceptions directly affect how they use and interpret accounting data (Kutluk, 2017). Human behavior is studied in the context of behavioral accounting to make sense of how it affects economic decision-making (Kutluk, 2017). In behavioral accounting research, people’s attitudes and behaviors are examined in order to understand how they are influenced by accounting events during their economic decision-making process (Kutluk, 2017). It is one of the main focuses of behavioral accounting research to apply behavioral science to understand and forecast human behavior wherever it is possible. The findings of our study indicate that REBT-based business coaching improves market traders’ perception of risk and their ability to manage credit risk, which makes it important that accounting professionals study the relationship between the empirical assumptions of REBT-based business coaching and economic decision-making in various business contexts. The business coaching and accounting professions should also investigate how REBT-based business coaching can also be used to improve perceptions, ethical orientations, attitudes, values, and behaviors of other clients, such as entrepreneurs, prospective investors, bankers, online traders, corporate employees, and managers. In addition, business coaching and accounting professionals should use the empirical assumptions of REBT-based coaching to examine how workplace values, culture, beliefs, and orientations impact their ability to cope and make economic decisions in various organizational settings.

## Conclusion

Among Nigerian traders, the REBT-based business coaching program has proven to be particularly helpful in improving financial risk perception and credit risk management. Through seminars and workshops, the researchers recommend that Nigerian traders learn about the efficiency of REBT-based business coaching programs in enhancing their financial risk perception and credit risk management. In this way, proper financial management guidance can be provided to various sets of investors while using REBT-based business coaching. Business coaches and accounting professionals should reposition their coaching encounters with Nigerian traders by using REBT-based business coaching.

## Data Availability Statement

The datasets presented in this study can be found in online repositories. The names of the repository/repositories and accession number(s) can be found in the article/supplementary material.

## Ethics Statement

The studies involving human participants were reviewed and approved by Research Ethics Committee at the authors’ institution. The patients/participants provided their written informed consent to participate in this study.

## Author Contributions

All authors conceived the study, were equally responsible for the study design and implementation, agreed to be accountable for the content of the work, and approved the submitted version.

## Conflict of Interest

The authors declare that the research was conducted in the absence of any commercial or financial relationships that could be construed as a potential conflict of interest.

## Publisher’s Note

All claims expressed in this article are solely those of the authors and do not necessarily represent those of their affiliated organizations, or those of the publisher, the editors and the reviewers. Any product that may be evaluated in this article, or claim that may be made by its manufacturer, is not guaranteed or endorsed by the publisher.
